# Development of an optimized AAV2/5 gene therapy vector for Leber congenital amaurosis owing to defects in RPE65

**DOI:** 10.1038/gt.2016.66

**Published:** 2016-09-22

**Authors:** A Georgiadis, Y Duran, J Ribeiro, L Abelleira-Hervas, S J Robbie, B Sünkel-Laing, S Fourali, A Gonzalez-Cordero, E Cristante, M Michaelides, J W B Bainbridge, A J Smith, R R Ali

**Affiliations:** 1 Department of Genetics, UCL Institute of Ophthalmology, London, EC1V 9EL UK; 2 NIHR Biomedical Research Centre at Moorfields Eye Hospital, London, EC1V 2PD UK

**Keywords:** Genetic vectors, Neurological disorders

## Abstract

Leber congenital amaurosis is a group of inherited retinal dystrophies that cause severe sight impairment in childhood; RPE65-deficiency causes impaired rod photoreceptor function from birth and progressive impairment of cone photoreceptor function associated with retinal degeneration. In animal models of RPE65 deficiency, subretinal injection of recombinant adeno-associated virus (AAV) 2/2 vectors carrying *RPE65* cDNA improves rod photoreceptor function, and intervention at an early stage of disease provides sustained benefit by protecting cone photoreceptors against retinal degeneration. In affected humans, administration of these vectors has resulted to date in relatively modest improvements in photoreceptor function, even when retinal degeneration is comparatively mild, and the duration of benefit is limited by progressive retinal degeneration. We conclude that the demand for RPE65 in humans is not fully met by current vectors, and predict that a more powerful vector will provide more durable benefit. With this aim we have modified the original AAV2/2 vector to generate AAV2/5-OPTIRPE65. The new configuration consists of an AAV vector serotype 5 carrying an optimized *hRPE65* promoter and a codon-optimized *hRPE65* gene. In mice, AAV2/5-OPTIRPE65 is at least 300-fold more potent than our original AAV2/2 vector.

**Supplementary information:**

The online version of this article (doi:10.1038/gt.2016.66) contains supplementary material, which is available to authorized users.

## Introduction

Leber congenital amaurosis (LCA) is a group of inherited retinal dystrophies that cause severe sight impairment in childhood.^1^ Mutations in the gene encoding the 65 kDa retinal pigment epithelium–specific retinoid isomerase (RPE65) (locus name LCA2; OMIM #204100) are responsible in upto 10% of affected individuals. The encoded retinoid isomerase converts all-*trans* retinyl ester to 11-*cis* retinal, which is critical for regeneration of visual pigment in rod photoreceptor cells following exposure to light. RPE65 deficiency causes severe dysfunction of rod photoreceptor cells, which are wholly reliant on retinal pigment epithelium–derived RPE65, resulting in severely impaired night vision from birth. The function of cone photoreceptor cells, which mediate daylight vision, colour discrimination and high visual acuity, is relatively preserved in childhood because cones have access to an alternative source of 11-*cis* retinal.^2^ However, progressive degeneration of the outer retina associated with local accumulation of toxic retinyl esters^3^ results in progressively severe impairment of cone photoreceptor-mediated daylight vision by early adulthood.

In animal models of Rpe65 deficiency, delivery of *RPE65* cDNA using recombinant AAV2/2 vectors injected subretinally mediates dose-dependent improvements in retinal and visual function, as assessed by electroretinography (ERG) and vision-guided behaviour^4, 5, 6^ respectively. When administered at an early stage of disease, protection against retinal degeneration results in sustained benefit in mice and dogs.^4, 7^ In humans, gene-augmentation therapy for RPE65 deficiency using AAV2/2 vectors can improve aspects of sight.^8, 9, 10, 11, 12^ However, improvements in photoreceptor function in humans have been relatively modest compared with those in animal models, even when retinal degeneration is relatively mild. Moreover, the duration of benefit to retinal sensitivity is limited by progressive retinal degeneration.^6, 13, 14^ Reports of more stable improvements in visual function have been based on the more subjective outcome measures of visual acuity, Goldmann perimetry and navigational vision) and pupilometry that is quantitative over only a limited range. We have found that *RPE65* expression in humans is greater than that in dogs,^8^ indicating a higher demand for RPE65 protein in the human retina. We conclude that the demand for RPE65 protein in humans is not fully met by current vectors and that greater, more durable benefit requires greater provision of RPE65 at an appropriately early point in disease progression.

Although intraocular administration of our original vector rAAV2/2.hRPE65p.hRPE65 (AAV2/2-hRPE65) was generally well tolerated in humans, a minority of participants receiving the higher dose of 10^12^ viral genomes (vg) developed transient intraocular inflammation with immune responses to AAV2 indicating dose-limiting toxicity.^8^ With the aim of enhancing provision of RPE65 protein without exceeding the maximum tolerated vector dose we have modified our original rAAV2/2 vector to increase the efficiency of transduction, transcription and translation. Specifically, this included optimisation of the promoter sequence, inclusion of an exogenous intron, optimisation of the Kozak sequence, and codon optimisation of *RPE65* sequence. We selected an AAV2/5 vector because AAV2/5 vectors are more effective than AAV2/2 vectors for gene delivery to human RPE cells *in vitro.* In mice, the optimized vector AAV2/5-OPTIRPE65 improves retinal function with at least 300-fold greater potency. We hypothesize that, in humans affected by LCA2, AAV2/5-OPTIRPE65 will provide higher levels of RPE65 protein than AAV2/2-hRPE65, leading to greater and more sustained benefit.

## Results and discussion

**Figure 1:**
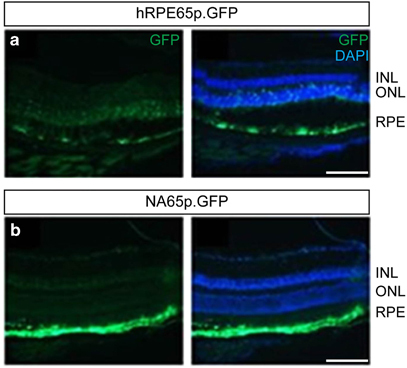
Optimized promoter (NA65p) drives expression of GFP with greater efficiency and with higher specificity for RPE cells. Representative GFP fluorescence in retinal cryosections of mice 4 weeks following subretinal injection of AAV2/8-hRPE65.GFP (**a**) or AAV2/8-NA65p.GFP (**b**). 4′,6-diamidino-2-phenylindole was used to visualize nuclei. GFP and merged images are shown for both panels. INL, inner nuclear layer; outer nuclear layer; RPE. Viral dose: 4 × 109 vg/eye, *n*=3. Scale bar, 60 μm.

**Figure 2:**
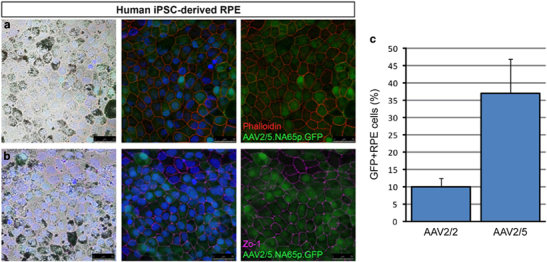
Transduction of human iPS-derived RPE cells with AAV2/5 vectors. RPE cells derived from human induced pluripotent stem (iPS) cells were transduced using AAV2/5-NA65p.GFP. Phalloidin (**a**) and ZO-1 (**b**) staining was used to visualize individual cells in the cultured RPE monolayer. 4′,6-diamidino-2-phenylindole was used to visualize nuclei. Light field, merged and merged without nuclear stain images are shown for both panels. Scale bar, 25 μm. (**c**) Comparison of human iPS-derived RPE transduction efficiencies of AAV2/2-CMV.GFP and AAV2/5-CMV.GFP suggests AAV2/5 more efficiently transduces human RPE cells, although the difference just failed to reach significance (*P*=0.058; mean±s.e.m.; *n*=3).

To enhance the specificity and efficiency of expression we modified the hRPE65 promoter by excising inhibitory elements of the promoter using naturally occurring restriction enzyme sites. We generated a range of fragment sizes based on the original hRPE65 promoter and selected a fragment from position −742 to +15 relative to the transcription start site. Following subretinal injection of AAV2/8 vectors into wild-type (WT) mice, we found that the optimized promoter (NA65p) led to a marked increase in expression of green fluorescent protein (GFP) in RPE cells (Figure 1), compared with the original promoter (hRPE65p). Furthermore, the optimized NA65p promoter conferred substantially greater specificity of expression for RPE cells, with no evidence of ectopic expression in photoreceptor cells (Figure 1). Comparison of GFP expression in the RPE driven by NA65p with that of the constitutive promoter CBA (or CAG) in mice *in vivo* was performed, following dissection of RPE/choroid from injected eyes and demonstrated significantly higher GFP messengerRNA (mRNA) levels driven by NA65p (*P*<0.001; Supplementary Figure S2). To confirm that the NA65p promoter drives expression in human cells we transduced RPE cells derived from human induced pluripotent stem (iPS cells) using AAV2/5-NA65p.GFP. Robust GFP fluorescence provided evidence of efficient AAV2/5-NA65p mediated expression in human cells expressing markers of differentiated RPE (Figures 2a and b). As AAV2/2 has proven to be a relatively inefficient serotype in various tissues, we compared the abilities of AAV2/2-CMV.GFP and AAV2/5-CMV.GFP to transduce human iPS cell derived RPE cells *in vitro.* The proportion of RPE cells positive for GFP was almost 4-fold greater after transduction with AAV2/5 (Figure 2c; Supplementary Figure S3 for representative images). Although this did not quite reach significance (*P*=0.058) it indicated that AAV2/5 was likely to be a more effective serotype for the transduction of human RPE.

**Figure 3:**
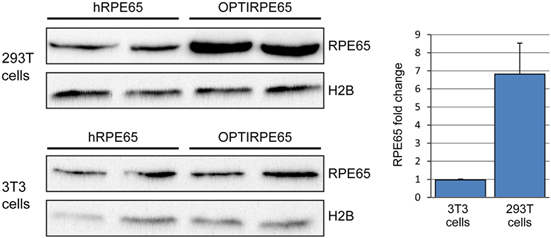
*In vitro* RPE65 protein production following transfection of human (293 T) and murine (3T3) cells with plasmids carrying wild-type *hRPE65* cDNA or codon-optimized *hRPE65* cDNA with SV40 intron. Western blot of whole cell lysates following transfection of pD10/CMV.hRPE65 plasmid (hRPE65) or pD10/CMV.OPTIRPE65 (OPTIRPE65) plasmid in human and mouse cells. 293 T and 3T3 cell lines were transfected with equal amounts of either pD10/CMV.hRPE65 or pD10/CMV.OPTIRPE65 plasmid and 10 μg of whole cell lysates were used for immunoblotting with a human RPE65 antibody (RPE65). Histone 2B (H2B) was used as a loading control. Quantification of RPE65 protein band intensities and comparison between hRPE65 and OPTIRPE65 for both 293 T and 3T3 cell lines. (*P*=0.04; mean±s.d.; *n*=2).

To enhance protein translation in human cells we generated an optimized cDNA sequence for the *hRPE65* transgene exploiting the degeneracy of the genetic code; intracellular availability of transfer ribonucleic acids specific for each amino acid varies, and optimising the *RPE65* coding sequence in favour of transfer ribonucleic acids that are more abundant in human cells can promote RPE65 protein translation in humans.^15^ We replaced 7 rare codons (including a pair in tandem), a cryptic splice site, 4 cryptic premature polyadenylation sites and a direct repeat of 50 basepairs, and we improved the codon usage frequency. To optimize gene expression further, we introduced an SV40 intron and an optimized Kozak sequence between the promoter and the *hRPE65* gene (Supplementary Figure S1). We compared the translation efficacy of the codon-optimized *RPE65* (OPTIRPE65) gene with the wild-type *RPE65* gene *in vitro* in human (HEK 293 T) and murine (3T3) cells by plasmid transfection, using a cytomegalovirus (CMV) promoter in each case since the human *RPE65* promoter is not active in these cell lines. Although no more efficient in murine 3T3 cells, in human HEK293T cells the OPTIRPE65 plasmid mediated significantly greater RPE65 protein production than the original RPE65 coding sequence as assessed by optical density measurements on western blots of whole cell lysates (OPTIRPE65 5.6±1.4 arbitrary units, hRPE65 0.79±0.039 arbitrary units, *P*=0.04; Figure 3). The increase in RPE65 protein production is not solely based on the codon optimisation of the RPE65 gene but also on the inclusion of the SV40 intron and Kozak sequence. The optimized plasmid construct provided improved mRNA stability and processing in human RPE cells *in vitro*, resulting in 2.5-fold greater levels of mRNA (Supplementary Figure S4). Since the impact of codon optimization for human tRNA is not evident in the murine cells the expression of the optimized construct in mice is likely to underestimate its potential efficiency of expression in humans.

**Figure 4:**
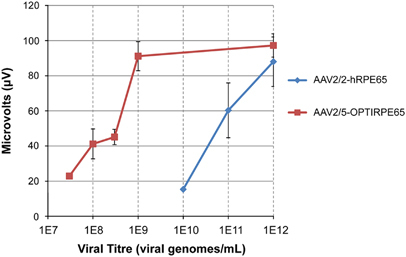
Dose-response of the AAV2/5-OPTIRPE65 on retinal function as demonstrated by electroretinography. Rpe65-deficient mice were injected with AAV2/5-OPTIRPE65 in one eye and with AAV2/2-hRPE65 in the contralateral eye at increasing titres. Retinal activity in the animals was assessed by dark-adapted electroretinography at 3 cd/m^2^. The graph shows average scotopic b-wave amplitudes (mean±s.d.) at 6 weeks post-treatment, when both vectors had reached peak expression. *n*=4.

To increase the efficiency of RPE cell transduction we packaged OPTIRPE65 into an AAV2/5 serotype vector and compared the effect of the optimized vector (AAV2/5-OPTIRPE65) with that of the original vector (AAV2/2-hRPE65) on electroretinography in the mouse model for LCA2 (*Rpe65*^−/−^). AAV2/5-OPTIRPE65 was tested at a concentration range of 3 × 10^7^ to 1 × 10^12^ vg per mL. AAV2/2-hRPE65 was tested at 1 × 10^10^ to 1 × 10^12^ vg per mL as concentrations lower than 1 × 10^10^ vg per mL showed no evidence of benefit Subretinal administration of AAV2/5-OPTIRPE65 at a 300-fold dilution resulted in mean scotopic b-wave responses at 6-weeks as great as, or greater than, responses following administration of the original vector (Figure 4). The ERG improvement achieved with AAV2/5-OPTIRPE65 at the 1 × 10^9^ concentration seems to reach a plateau of rescuable retinal function in the LCA2 mouse model, as similar levels of response were observed after administration at a titre of 1 × 10^12^ vg per mL (average amplitude 97±13 μV; Figure 4). WT mouse scotopic b-wave responses at a similar age are measured at 180–200 μV. This finding demonstrates that AAV2/5-OPTIRPE65 is at least 300-fold more potent than AAV2/2-hRPE65 in restoring rod photoreceptor responses to light stimulation. The potency of AAV2/5-OPTIRPE65 also compares favourably against AAV2/2.CBA vectors in RPE65-deficient mice: while AAV2/5-OPTIRPE65 at a dose of 4 × 10^6^ vg leads reliably to improved b-wave amplitudes (average amplitude 91±9 μV; Figure 4), published data suggest AAV2/2.CBA vectors at a dose of 1 × 10^9^ vg offer a less robust benefit (improvements in 4 out of 10 animals; average b-wave amplitude of 60 μV).^16^ The enhanced benefit of AAV2/5-OPTIRPE65 to retinal function is the consequence of the more rapid transduction kinetics of the AAV2/5 serotype and improved mRNA stability owing to inclusion of the intron in the optimized gene cassette. As previously noted, the assessment in mice does not reflect the additional advantage of codon optimisation for protein translation in human cells specifically, which confers no such advantage in mouse cells (Figure 3).

**Figure 5:**
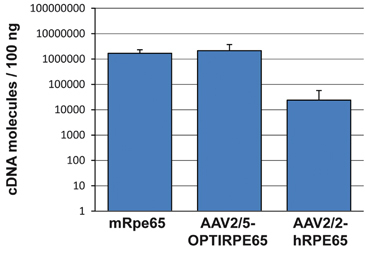
AAV2/5-OPTIRPE65 mediates physiological expression levels of *RPE65* mRNA in the mouse retina. Whole retina lysates from WT mice 6 weeks following injection with AAV2/5-OPTIRPE65 or AAV2/2-hRPE65 were used for quantitative real-time PCR (qPCR). Absolute quantification of total mRNA-derived cDNA was performed for human *RPE65* transcripts, *OptiRPE65* transcripts and endogenous murine *Rpe65* transcripts (mean±s.d.). Dose: 4 × 10^9^ vg per eye, *n*=4.

To compare the production of *OPTIRPE65* mRNA from AAV2/5-OPTIRPE65 and *hRPE65* mRNA from AAV2/2-hRPE65 with endogenous mouse *Rpe65* (*mRpe65*) mRNA, we performed absolute quantification using quantitative real-time PCR (qPCR) in retinal lysates of WT animals 6 weeks following intraocular vector administration at a dose of 4 × 10^9^ vg per eye (1 × 10^12^ vg per mL). Total cDNA was prepared from retinal lysates and the number of OPTIRPE65, human RPE65 and murine Rpe65 transcript molecules was measured in 100 ng. We found no significant difference between the number of OPTIRPE65 and endogenous murine Rpe65 transcripts (Figure 5). In contrast, AAV2/5-OPTIRPE65 produced >100-fold greater number of transcripts than AAV2/2-hRPE65 (*P*<0.05). This finding indicates that in mice *in vivo* the OPTIRPE65 expression cassette can mediate mRNA expression similar to the physiological levels of the murine *Rpe65* gene.

**Figure 6:**
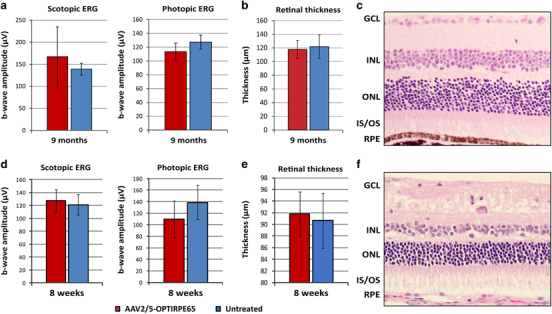
Structural and functional assessments of WT mouse and rabbit retinas following subretinal injection of AAV2/5-OPTIRPE65. ERG responses and retinal thickness measurements of eyes injected with AAV2/5-OPTIRPE65 (*n*=10 for mice, *n*=9 for rabbits) and untreated control eyes (*n*=4 for mice, *n*=9 for rabbits) of young adult WT mice. Mean±s.d. scotopic (at 0.01 Cds per m^2^) and photopic (at 10 Cds per m^2^) ERG b-wave amplitudes of mice retinas at 9 months (**a**) or rabbit retinas at 8 weeks (**d**) are shown. The *Y* axis denotes μV values of electrical conductivity. Dose: 4 × 10^9^ vg per eye in mice, 2 × 10^11^ vg per eye in rabbits. Mean±s.d. photoreceptor cell layer thickness measurements from treated and untreated eyes are shown (**b** for mice, **e** for rabbits). Representative images of mouse (**c**) and rabbit (**f**) retinal morphology: GCL, ganglion cell layer; INL, inner nuclear layer; outer nuclear layer; IS/OS, inner segments/outer segments; RPE.

To investigate the safety of long-term overproduction of RPE65 protein we measured retinal function and retinal thickness 9 months following subretinal administration of AAV2/5-OPTIRPE65 at a dose of 4 × 10^9^ vg per eye (1 × 10^12^ vg per mL) in young adult WT mice and 8 weeks after administration of 2 × 10^11^ vg per eye (1 × 10^12^ vg per mL) in young adult rabbits (Figure 6). This dose in mice is 1000-fold higher than the dose required for a restoration of electrophysiological responses in RPE65-deficient mice. Furthermore, the vector-mediated expression of human RPE65 protein is superimposed on the normal endogenous expression of mouse Rpe65 protein in WT animals. No significant difference in mean rod-mediated and cone mediated b-wave amplitudes was evident at 9 months in mice and at 8 weeks in rabbits (Figures 6a and d). On histological examination, we measured no difference in outer nuclear layer thickness in sections through the optic nerve, and observed normal preservation of retinal layering and photoreceptor nuclei (Figures 6b, c, e and f). We conclude that overexpression of RPE65 protein in the mouse and rabbit RPE is well tolerated.

Having identified evidence of systemic immune responses against AAV2/2 capsid and intraocular inflammation in a small minority of human participants following intraocular vector administration, we have since optimized our AAV manufacturing protocols to reduce the proportion of empty vector particles from 80 to 10%. The removal of empty vector particles is expected to minimize immune responses and preserve the availability of cell surface receptors for binding by filled vector particles. Since we found that exposure of OPTIRPE65 vector to the surfaces of the delivery syringe or cannula leads to no substantial reduction of the effective dose in RPE65-deficient mice, we have chosen not to supplement the recipients with a surfactant, as has been described by others.^9^

The surgical technique for vector administration is designed to target RPE cells underlying surviving photoreceptor cells as demonstrated structurally by OCT scanning and functionally by perimetry. Exploiting the natural plane of cleavage between the RPE and overlying photoreceptors, vector suspension is injected through the neurosensory retina via one or more small retinotomies into this potential space, causing separation of the cell layers. Active absorption of fluid by RPE cells promotes their transduction by vector particles, and the temporary retinal detachment typically resolves spontaneously within 48 h. Although even temporary detachment of the retina can adversely affect retinal structure and function, the relatively modest risk of harm to vision can be justified by the potential for benefit from restoration of retinoid metabolism. The extent of the retinal area targeted deserves careful consideration; given that non-cell-autonomous mechanisms can contribute to retinal cell death, widespread administration to surviving retina may help protect against degeneration. Although the sites for injection retinotomies can be precisely selected, the distribution of vector suspension within the subretinal space is strongly influenced by variable regional resistance to retinal detachment. Given that the balance of risks relating to subfoveal delivery of vector in RPE65-deficiency appears to be less favourable^8, 17^ delivery techniques that minimize the height and duration of any foveal detachment may be desirable, possibly through the use of multiple injection retinotomies.

AAV2/5-OPTIRPE65 improves retinal function *in vivo* in mice at titres around 300-fold lower than the original AAV2/2-hRPE65 vector. The impact of codon optimisation for human cells is expected to improve this 6-fold further in humans, providing up to 1800-fold greater potency. When compared with an AAV2/2-CBA.hRPE65 vector, cautious estimates of differences suggest that the AAV2/5-OPTIRPE65 vector uses a promoter as effective as the CBA promoter, a coding sequence 7-fold more effective, an AAV serotype about 4-fold more effective (Figure 2c) and a vector dose 6-fold higher. As these relative efficacies are independent, the overall difference in RPE65 production can be conservatively estimated at 150-fold greater efficiency than an AAV2/2 vector carrying *hRPE65* driven by a CBA promoter. We hypothesize that, in humans affected by LCA2, AAV2/5-OPTIRPE65 will provide higher levels of RPE65 protein than either AAV2/2-hRPE65 or AAV2/2.CBA.hRPE65, leading to greater and more sustained benefit.

## Materials and methods

### Cell lines, plasmid transfection and viral transduction

Mouse fibroblasts (3T3) and human embryonic kidney cells (293Ts) were maintained with DMEM medium supplemented with 10% fetal bovine serum. The human embryonic stem cell line H9 and induced pluripotent stem cell line IRM90-4 (Wicell) were maintained under feeder free conditions on E8 and geltrex coated six well plates and tested for mycoplasma infection monthly. Differentiation of stem cells to RPE cell fate was achieved using a multi-step protocol adapted from previous published protocols^18, 19^ (Supplementary Methods). For plasmid transfections, 100 000 cells were plated the day before on 24 well plates. One microgram of corresponding plasmids was transfected using Lipofectamine 2000 according to manufacturer’s directions (Life Technologies, Paisley, UK) and cells were analysed 48 h after transfection. For transduction of ~2 × 10^6^ iPS-derived RPE cells, 1.2 × 10^11^ vg of AAV2/5-NA65p.GFP, AAV2/5-CMV.GFP or AAV2/2-CMV.GFP were added to each well.

### Virus preparation

AAV backbone plasmid constructs were packaged into AAV2, AAV5 and AAV8 to generate the recombinant AAV viral vectors required for this study (Supplementary Figure.S1). Recombinant vectors were produced through a triple transient transfection method using published methods^20^ (Supplementary methods).

### Animals and subretinal injections

The *Rpe65* knockout mice (*Rpe65*^*rd12/rd12*^) were purchased from Jackson Laboratories (US) and WT mice (C57BL/6J) were purchased from Harlan Laboratories (Blackthorn, UK). WT rabbits (New Zealand Whites) were purchased from Envigo (Hundingdon, UK). Young adult (6–10 weeks) animals were used throughout. WT animals were all females, while *Rpe65*^*−/−*^ were mixed sex. Animals were maintained under cyclic light (12 h light–dark) conditions. All experiments were approved by the local Institutional Animal Care and Use Committees (UCL, London, UK) and conformed to the guidelines on the care and use of animals adopted by the Society for Neuroscience and the Association for Research in Vision and Ophthalmology (Rockville, MD, USA). Subretinal administration of vectors was performed as previously described^20^ (Supplementary methods). Eyes were assigned as treated and (contralateral) control eyes using randomisation software (https://www.randomizer.org/). After the injection procedure, investigators were masked regarding the eye receiving treatment during the *in vivo* stages. Four eyes from 4 animals were treated for efficacy assessments, based on a power calculation to detect a minimum 10-fold difference in potency.

### Immunostaining

iPS-derived RPE cells were fixed on 4% Paraformaldehyde for immunocytochemistry analysis 7 days post virus transduction. Cells were blocked in 5% goat serum and 1% bovine serum albumin in PBS containing 0.1% Triton-X100. Primary rabbit anti-ZO-1 antibody (Life technologies, Paisley, UK) was used at 1:200 and rabbit anti-phalloidin TRITC (Sigma-Aldrich, Dorset, UK) was used at a concentration of 1:500. Primary antibodies were incubated overnight at 4 °C in blocking solution. Secondary antibody staining was performed for 2 h at room temperature followed by PBS washing and counterstaining with 4′,6-diamidino-2-phenylindole.

### RNA extractions and cDNA preparation

Enucleated eyes (Figures 1 and 5), RPE/choroid dissections (Supplementary Figure.S2) or cell pellets (Figure 3, Supplementary Figure S4) were obtained and total mRNA was extracted using an RNeasy mini Kit (Qiagen, Crawley, UK). One microgram of total RNA was used to generate cDNA using the QuantiTect Reverse Transcription kit (Qiagen, Crawley, UK). According to manufacturer’s directions, the conversion of RNA to cDNA should be at a rate of 1:1.

### qPCR

Real-time quantitative RT-PCR (qPCR) was performed with a commercial thermal cycler (7900HT; Applied Biosciences, Foster City, CA). All reagents were obtained from Roche Diagnostics (Burgess Hill, UK). The technique was based on FAM-labelled hydrolysis probes (Roche Diagnostics, Burgess Hill, UK), and primers were designed for specific probe-binding regions using the Roche Universal Probe Library (see Supplementary Methods for details). For absolute quantification of murine, human and human codon-optimized RPE65 genes, amplicons specific to each reaction were used to prepare serial dilution standard curves ranging from 10^9^ to 10^2^ molecules. The standard curve was used for sample molecule number interpolation.

### Electroretinographic analysis

ERGs were recorded from both eyes of Rpe65^−/−^ mice, C57BL6/J WT mice and New Zealand White WT rabbits. All animals were dark adapted overnight before ERG recordings under anaesthesia. The pupils were dilated with a drop of Minims Tropicamide 1% (Bausch & Lomb/Chauvin Pharmaceuticals, Essex, UK). Midline subdermal ground and mouth reference electrodes were first placed, followed by eye electrodes that were allowed to lightly touch the corneas. A drop of Viscotears 0.2% liquid gel (Dr Robert Winzer Pharma/OPD Laboratories, Watford, UK) was placed on top of the electrodes to keep the corneas moistened during recordings. Scotopic and photopic ERGs were recorded with commercially available equipment (Espion E2; Diagnosys, Lowell, MA) using published methods^20^ (see Supplementary Methods for details).

### Statistical analysis

Experimental results were analysed using Student *t*-test (Figures 2, 3 and 6 and S2) or One-way analysis of variance (Figure 5), including D’Agostino and Pearson normality tests and Bartlett’s test for equal variance. All analyses were performed 2-sided. Graphs show means±s.e.m. N-numbers and *P*-values are reported in the figure legends. N-numbers were chosen, based on power calculations. No animals or samples were excluded from analysis.

## Supplementary information

Supplementary Informations (PDF 738 kb)
