# Correction: Development of an optimized AAV2/5 gene therapy vector for Leber congenital amaurosis owing to defects in RPE65

**DOI:** 10.1038/s41434-018-0031-x

**Published:** 2018-07-25

**Authors:** A. Georgiadis, Y. Duran, J. Ribeiro, L. Abelleira-Hervas, S. J. Robbie, B. Sünkel-Laing, S. Fourali, A. Gonzalez-Cordero, E. Cristante, M. Michaelides, J. W. B. Bainbridge, A. J. Smith, R. R. Ali

**Affiliations:** 1 Department of Genetics, UCL Institute of Ophthalmology, London, EC1V 9EL UK; 2 NIHR Biomedical Research Centre at Moorfields Eye Hospital, London, EC1V 2PD UK

Correction to: *Gene Therapy* 10.1038/gt.2016.66, published online 22 September 2016

This Article was originally published under a CC BY-NC-SA 4.0 license, but has now been made available under a CC BY 4.0 license. The PDF and HTML versions of the Article have been modified accordingly.

