# Supplementary material for: Development of an optimized AAV2/5 gene therapy vector for Leber congenital amaurosis owing to defects in RPE65
Source: Gene Ther. 2016 Sep 22;23(12):857–62. doi: 10.1038/gt.2016.66 (PMC5143366; doi:10.1038/gt.2016.66)
Supplement: Supplementary file 1 — Supplementary Informations (PDF 738 kb) [file 41434_2016_BFgt201666_MOESM7_ESM.pdf]

Supplementary Figures

Figure S1. Schematic diagram of key constructs

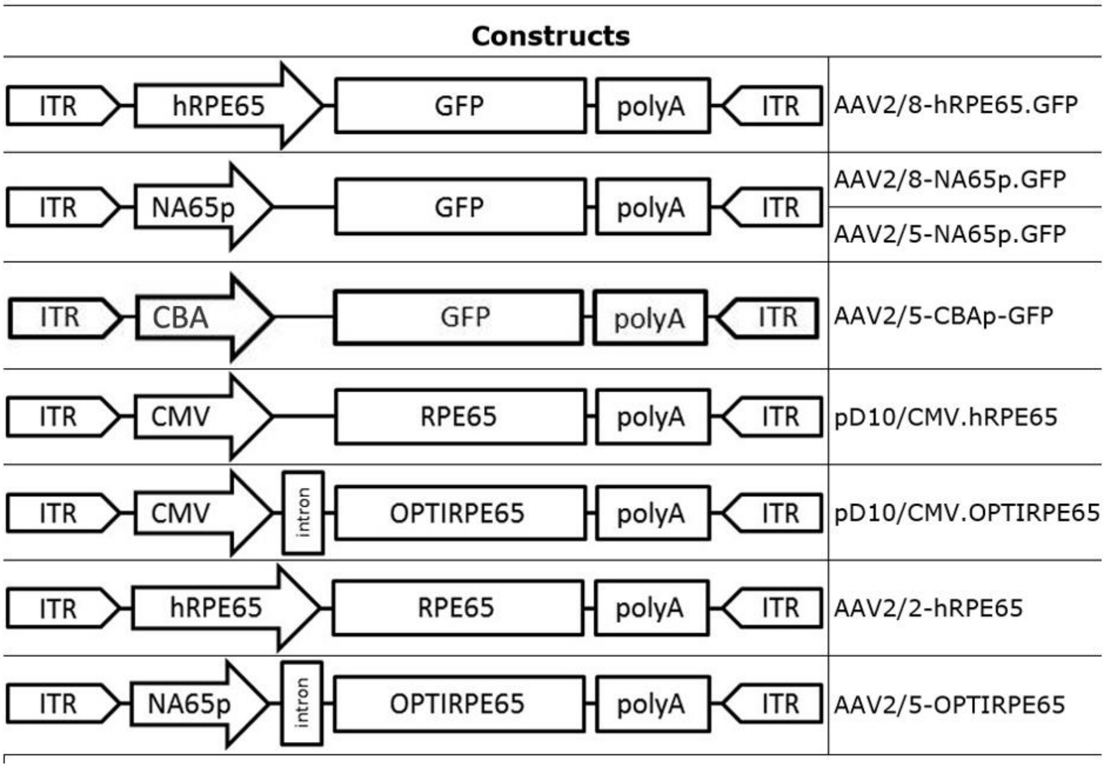

Schematic description of gene expression constructs used in this study either in plasmid form (pD10) or as an AAV vector (AAV). ITR; AAV2 inverted terminal repeats. CMV; Cytomegalovirus promoter. CBA; Chicken beta-actin promoter. hRPE65; human RPE65 promoter. NA65p; optimized human RPE65 promoter. Intron; SV40 intron sequence. GFP; Green fluorescent protein. RPE65; human RPE65 gene. OPTIRPE65; codon-optimised human RPE65 gene. PolyA; SV40 polyadenylation signal.

Figure S2. Relative quantification of mRNA expression driven by NA65 promoter (NA65p) and CBA promoter (CBAp) *in vivo* in mouse RPE monolayer

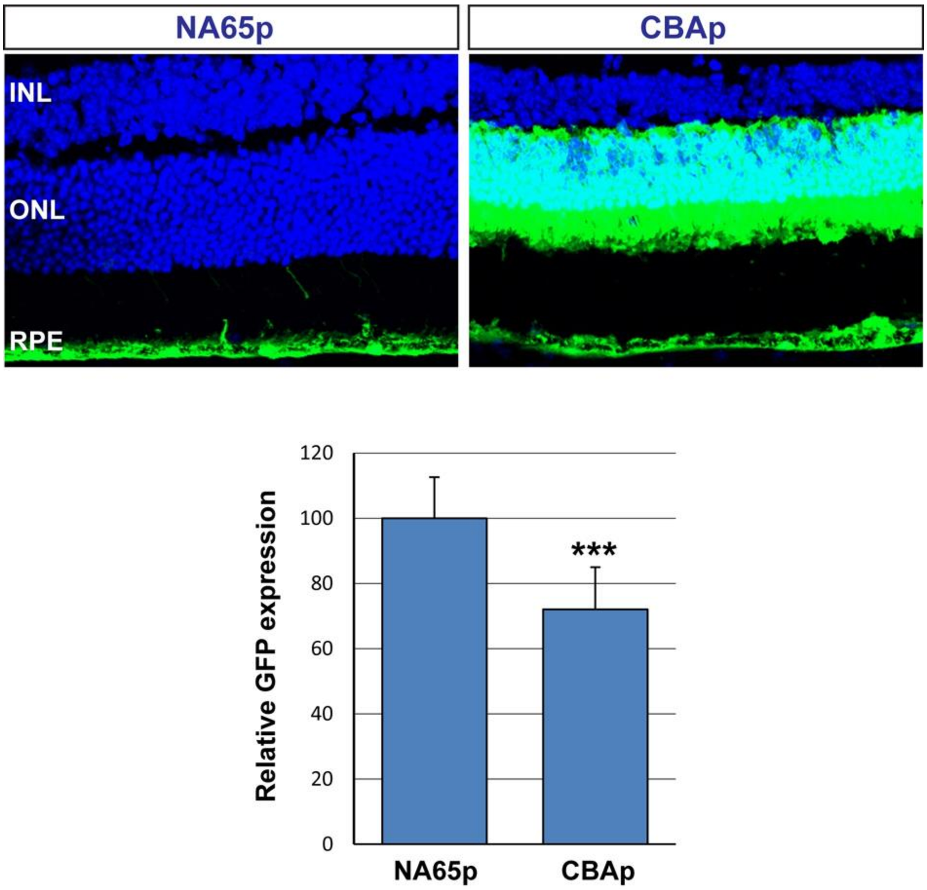

AAV2/5-NA65p.GFP and AAV2/5-CBAp.GFP viruses were injected in contralateral eyes in wild-type mice. After 6 weeks of administration, eyes were sectioned and GFP was visualised in sections from AAV2/5-NA65p.GFP (NA65p) and AAV2/5-CBAp.GFP (CBAp) injected eyes. DAPI was used to visualise nuclei. Relative quantification by qPCR was performed on RPE/choroidal dissections following mRNA extraction. mRpe65 was used for normalisation. Bars indicate mean ± SD. P=0.0008; n=4.

Figure S3. Transduction of human iPS-derived RPE cells with AAV2/2-CMV.GFP and AAV2/5-CMV.GFP vectors

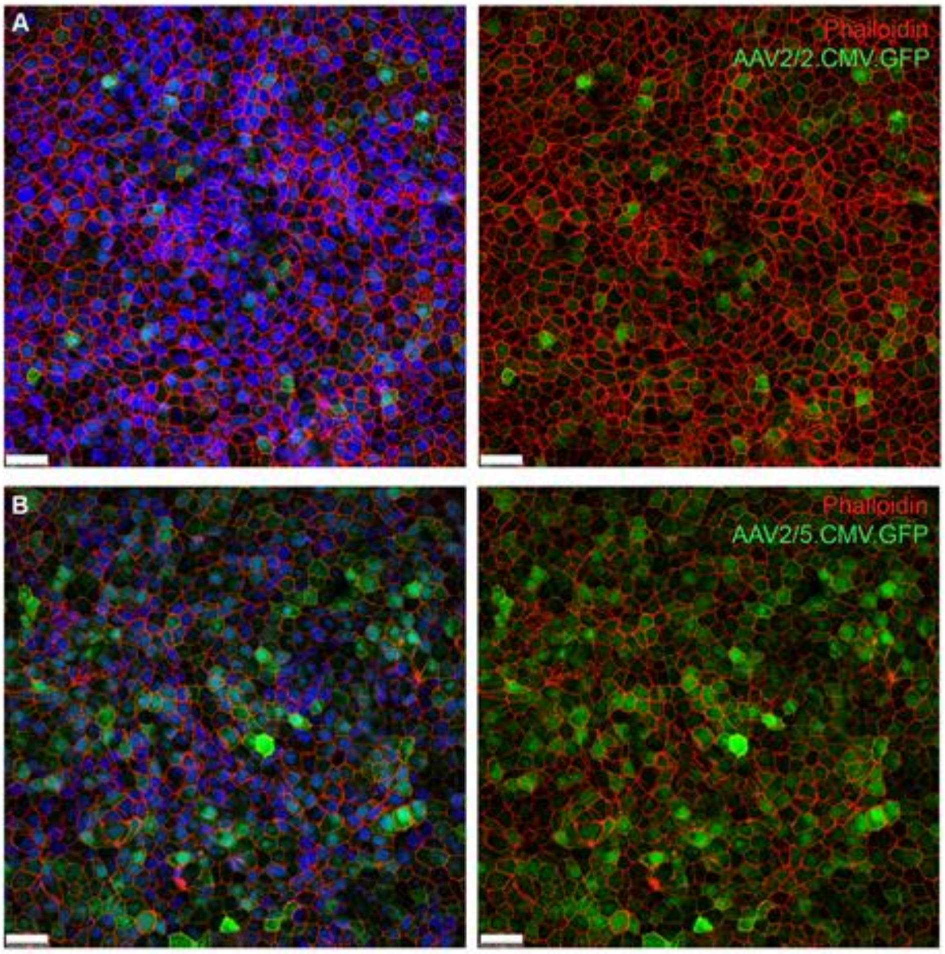

RPE cells derived from human induced pluripotent stem (iPS) cells were transduced using either AAV2/2-CMV.GFP (A) or AAV2/5-CMV.GFP (B). Phalloidin staining was used to visualise individual cells in the cultured RPE monolayer and DAPI was used to visualise nuclei. Phalloidin, GFP and DAPI merged images (left panels) and Phalloidin and GFP only (right panels) are shown. Scale bar; 25 µm.

Figure S4. Quantification of *RPE65* expression *in vitro* in human RPE cells transfected with plasmids carrying wild type (CMV.hRPE65) or codon-optimised hRPE65 cDNA with SV40 intron (CMV.OPTIRPE65)

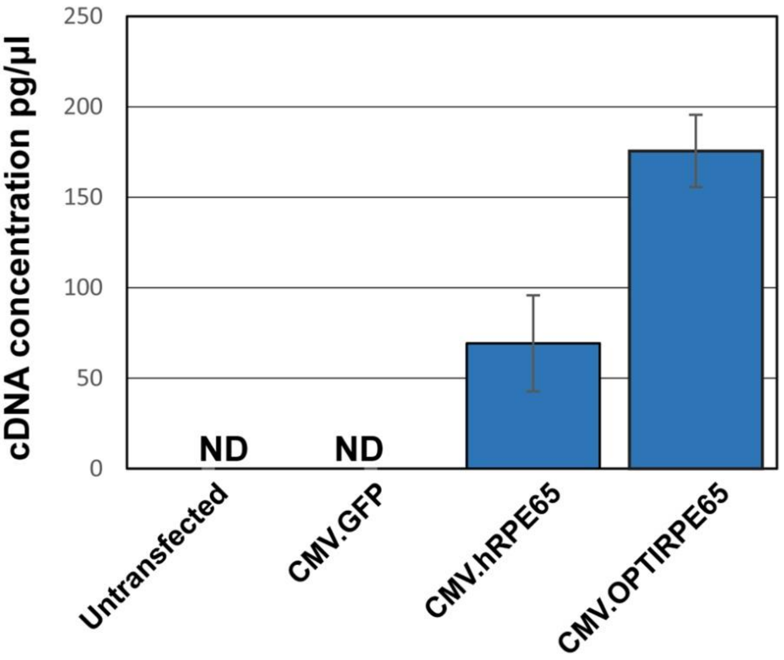

RPE cells derived from human embryonic stem (ES) cells were transfected with pD10/CMV.hRPE65 (CMV.hRPE65) or pD10/CMV.OPTIRPE65 (CMV.OPTIRPE65). Absolute quantification of total mRNA-derived cDNA was performed for both OptiRPE65 and human RPE65 genes. Untransfected and GFP-transfected control (CMV.eGFP) cells were also used. Values indicate mean ± SD. n=3.

Supplementary Methods

Virus preparation and titre

Recombinant vectors were produced through a triple transient transfection method. The plasmid construct, AAV serotype-specific packaging plasmid and helper plasmid, in a ratio of 1:1:3 at 20 mg total DNA per ml of DMEM, were mixed with Polyethylenimine (Polysciences Inc.) to a final concentration of 50 mg.mL^{-1} and incubated for 10 min at room temperature to form transfection complexes that were added to 293T cells at 50 µg DNA per 15-cm plate and left for 72 h. The cells were collected, concentrated and lysed by freeze–thaw (3x) in PBS to release the vector. Recombinant AAV was bound to an AVB Sepharose column (GE Healthcare), and eluted with 50mM Glycine pH2.7 into 1M Tris pH 8.8. Vectors were washed in 1x PBS and concentrated to a volume of 100–150 µl using Vivaspin 4 (10 kDa) concentrators. Viral particle titres were determined by quantitative real-time PCR (qPCR) using an ITR binding assay as previously described (Aurnhammer et al, 2012).

Subretinal injections

Mice were anesthetised with a single intraperitoneal injection of a 0.01-ml/g mixture of Domitor (1 mg/ml medetomidine hydrochloride), ketamine (100 mg/ml), and water at a ratio of 5:3:42 before surgical procedures. Rabbits were anesthetised with a single intraperitoneal injection of a 0.5-ml/kg mixture of Domitor (1 mg/ml medetomidine hydrochloride) and 0.25ml/kg ketamine (100 mg/ml). Subretinal injections were performed under direct retinoscopy thorough an operating microscope. The tip of a 1.5-cm, 34-gauge hypodermic needle (Hamilton) was inserted tangentially through the sclera of the mouse eye, causing a self-sealing wound tunnel. A 30-gauge needle was used for injections into rabbit eyes. The needle tip was brought into focus between the retina and retinal pigment. Mice received double injections of 2 µl each to produce bullous retinal detachments in the superior and inferior hemisphere around the injection sites. Rabbits received one injection of 200 µl in the superior hemisphere. Eyes were assigned as treated and (contralateral) control eyes using randomisation software.

qPCR primers

The primers used for murine Rpe65 were TCAGGAGATATGTACTTCCTTTGACA (Forward) and TTGTATGGGGCAGTGTGACT (Reverse). The primers used for human RPE65 were CAATGGGTTTCTGATTGTGGA (Forward) and CCAGTTCTCACGTAAATTGGCTA (Reverse). The primers used for codon-optimised human RPE65 (OPTIRPE65) were CAACTATCTGTACCTGGCAAACC (Forward) and GGTGCTTTTCGAGCATTTTT (Reverse). The primers used for murine beta-actin were AAGGCCAACCGTGAAAAGAT (Forward) and GTGGTACGACCAGAGGCATAC (Reverse). The primers used for green fluorescent protein were GAAGCGCGATGACATGGT (Forward) and CCATGCCGAGAGTGATCC (Reverse).

Western Blot of cellular protein extracts

Protein extracts were quantified in a Bio-Rad protein assay (Bio-Rad, Hemel Hempstead, UK). Equal amounts of each sample were loaded onto 6% polyacrylamide gels and, after gel electrophoresis,

transferred to nitrocellulose membrane for Western blotting. The membrane was cut in using the loading ladder as guide to separate parts of the membrane according to blotted proteins (RPE65 or Histone 2B). After blocking the membrane in block solution (5% skimmed milk powder–1% bovine serum albumin [BSA; Sigma-Aldrich]–0.05% Tween 20 in PBS) overnight at 4°C, mouse monoclonal anti-RPE65 antibody [diluted 1:1000; Ab13826, Abcam, Cambridge, UK] or mouse monoclonal anti-H2B antibody [diluted 1:5000; 2934S, New England Biolabs, Wilbury, UK] was added and left overnight at 4°C. Membrane was thoroughly washed in PBS–0.05% Tween 20 and incubated with a horseradish peroxidase (HRP)-conjugated goat anti-mouse IgG antibody (diluted 1:5000; Dako, Cambridge, UK) in block solution for 1 hr at room temperature. After PBS–0.05% Tween 20 washes, signal was developed with Amersham enhanced chemiluminescence (ECL) Plus Western blotting detection reagents (GE Healthcare, Chalfont St Giles, UK).

Electroretinographic analysis

Electroretinograms (ERGs) were recorded from both eyes of Rpe65-/- mice, C57BL6/J wildtype mice and New Zealand White wild-type rabbits. All animals were dark adapted overnight before ERG recordings. Following anaesthesia, the pupils were dilated with a drop of Minims Tropicamide 1% (Bausch & Lomb/Chauvin Pharmaceuticals, Essex, UK). Midline subdermal ground and mouth reference electrodes were first placed, followed by eye electrodes that were allowed to lightly touch the corneas. A drop of Viscotears 0.2% liquid gel (Dr. Robert Winzer Pharma/OPD Laboratories, Watford, UK) was placed on top of the electrodes to keep the corneas moistened during recordings. ERGs were recorded with commercially available equipment (Espion E2; Diagnosys, Lowell, MA). Bandpass filter cutoff frequencies were 0.312 Hz (low-frequency cutoff) and 1000 Hz (high-frequency cutoff). Scotopic, rod-mediated responses were obtained from dark-adapted animals at the following increasing light intensities: 0.001, 0.01, 0.1, 1 and 3 cds/m^2. Ten responses per intensity were recorded for the first three intensities with 10-sec dark adaptation between each. Five responses were recorded for all the subsequent steps with 30-sec dark adaptation between each. Responses were averaged for each intensity. Photopic, cone-mediated responses were performed after a 10-min light adaptation on a background light intensity of 30 cd/m^2, which was used as background intensity for the duration of photopic recordings, flash and flicker. Recordings were obtained at the following increasing light intensities: 0.1, 1, 3, 5 and 10cds/m^2. Twenty-five responses were averaged for each intensity, with a 60-sec light adaptation interval between each step.

Histology

Animals were killed, the eyes enucleated and the cornea, lens and iris removed. For retinal sections, eyecups were fixed in 4% paraformaldehyde for 1 h and incubated in 20% sucrose for 1 h at room temperature, before embedding in optimal cutting temperature medium. Twelve µm cryosections were cut in sagittal orientation, counterstained with 4’,6-diamidino-2-phenylindole (DAPI) and mounted with DAKO fluorescent mounting media (DAKO, S3023, Denmark). Images were acquired by confocal microscopy (Leica DM5500Q).

Human ESC maintenance and retinal differentiation culture

The human embryonic stem cell line H9 and induced pluripotent stem cell line IRM90-4 (Wicell) were maintained on feeder free conditions on E8 and geltrex coated 6 well plates. Stem cells were dissociated using a dispase and collagenase solution. Cell clumps were collected and resuspended in E8 media. The cell clumps were transferred to a low-binding 10 cm plate to form floating EBs. Differentiation media was changed to EB media (DMEM/F12 (1:1), Knockout serum replacement, MEM non-essential amino acids, L-glutamine, 2-Mercaptoethanol, Pen/Strep) containing 100 ng/ml of dorsomorphin (BMP inhibitor) and 100 ng/ml of XAV939 (DKK1-Wnt inhibitor) from day 2 to 3 of culture. From day 4 of culture media was changed to neural induction media (Advanced DMEM/F12, MEM non-essential amino acids, N2, Heparin and Pen/Strep). On day 7 EBs were plated on laminin for growth as adherent cultures. From day 15 media was changed to retinal maturation media (DMEM, F12, B27 without retinoic acid and Pen/Strep) containing 50 ng/ml of Activin A (R&D) was added from day 18 to 40. Islands of pigmented RPE appeared between day 40-60 and these regions were manually dissected for further expansion and quantification on laminin coated wells.

Aurnhammer,C., Haase,M., Muether,N., Hausl,M., Rauschhuber,C., Huber,I., Nitschko,H., Busch,U., Sing,A., Ehrhardt,A., and Baiker,A. (2012). Universal real-time PCR for the detection and quantification of adeno-associated virus serotype 2-derived inverted terminal repeat sequences. Hum. Gene Ther. Methods *23*, 18-28.
